# Reviewer Acknowledgements

**DOI:** 10.1186/1471-2407-13-186

**Published:** 2013-06-05

**Authors:** Christna Chap

**Affiliations:** 1 BioMed Central, Floor 6, 236 Gray's Inn Road London, WC1X 8HB United Kingdom

## Abstract

**Contributing reviewers:**

The editors of *BMC Cancer* would like to thank all our reviewers who have contributed to the journal in Volume 12 (2012).

## 

Trond Aasen

Spain

Abbas Abbas

USA

Ahmed Abdulamir

Malaysia

Filippo Acconcia

Italy

B.R Achyut

USA

Yasushi Adachi

Japan

Lucile L Adams-Campbell

USA

Christina Addison

Canada

Abbas Agaimy

Germany

Sofia Agelaki

Greece

Claudia Agnoli

Italy

Linda Agolli

Italy

Patrizia Agostinis

Belgium

Jose M Aguirre-Urizar

Spain

Tanya Agurs-Collins

USA

Dinesh Kumar Ahirwar

USA

Atique Ahmed

USA

Yong Chan Ahn

Korea, South

Nita Ahuja

USA

Wu Ai-Wen

China

Masaki Aizawa

Japan

Rosemary Akhurst

USA

Yoshihiro Akimoto

Japan

Salah-Eddin Al-Batran

Germany

Agustí Alentorn

Spain

Alex Alfieri

Germany

Elizabeth Algar

Australia

Juan Alguacil

Spain

Malcolm Alison

United Kingdom

Madson Almeida

Brazil

Michael Amatangelo

USA

Maria Raffaella Ambrosio

Italy

Stefan Ambs

USA

Masoud Amiri

Netherlands

Ole Ammerpohl

Germany

Ioannis Anagnostopoulos

Germany

Venkateshwari Ananthapur

India

Sudharsana Ande

Canada

Ladislav Andera

Czech Republic

Jesper Andersen

USA

Kristin Andersen

Norway

Christopher Anderson

USA

Ravindran Ankathil

Malaysia

Marc Antonyak

USA

Maarit Anttila

Finland

Hajer Aounallah-Skhiri

Tunisia

Keishiro Aoyagi

Japan

Hidefumi Aoyama

Japan

Nadezda Apostolova

Spain

Valentina Appierto

Italy

Nobuhito Araki

Japan

Raj Aravalli

USA

Luca Arcaini

Italy

Paolo Arcari

Italy

M.Isabel Arenas

Spain

Douglas Arenberg

USA

Jose-Antonio Arias-Montano

Mexico

Takaaki Arigami

Japan

Myron Arlen

USA

Ralph Arlinghaus

USA

Shanna Arnold

USA

Juan Ignacio Arraras

Spain

Rosa Artells

Spain

Hassan Ashktorab

USA

Sofia Asioli

Italy

Davidson Ateh

United Kingdom

Didier Attaix

France

Gerges Attia

Egypt

Paolo Aurello

Italy

Vicky Avery

Australia

Dror Avni

Israel

Koichi Azuma

Japan

Peter Baade

Australia

Hideo Baba

Japan

Hagen Bachmann

Germany

Claudia Backsch

Germany

Victoria Bae-Jump

USA

Claude Bagnis

France

Marina Bagnoli

Italy

Lea Baider

Israel

Joffre Baker

United Kingdom

Ovidiu Balacescu

Romania

Claudia Baldus

Germany

Justin Balko

USA

Debabrata Banerjee

USA

Yixi Bao

China

Sophie Barillé-Nion

France

Elizabeth Barnes

Canada

Joanne Barnes Weidhaas

USA

Ravi Barod

United Kingdom

Valeria Barresi

Italy

Kathryn Barry

USA

John Bartlett

Canada

Elisabeth Barton

USA

Matthias Barton

Switzerland

Katarina Bartuma

Sweden

Murali Bashyam

India

Mark Basik

Canada

Esther Bastiaannet

Netherlands

Eduard Batlle

Spain

Claudio Battaglini

USA

Da-Tian Bau

Taiwan

Michael Baudis

Switzerland

Kathrin Bauer

Germany

Freerk Baumann

Germany

Shrujal Baxi

USA

David Beer

USA

Bruce Bejcek

USA

Ruud Bekkers

Netherlands

Antonino Belfiore

Italy

Diana Bell

USA

Jeff Bell

USA

Saverio Bellizzi

Italy

Mariaserena Benassi

Italy

Katia Bencardino

Italy

Anna M Bennet

Sweden

Charlotte Benson

United Kingdom

Giovanni Luca Beretta

Italy

Anna Bergamaschi

USA

Corinna Bergelt

Germany

Ann Berger

USA

Gregor Berger

Switzerland

Walter Berger

Austria

Leanne Berkahn

New Zealand

Hans-Ulrich Bernard

USA

Vagner Bernardo

Brazil

Franco Berrino

Italy

J. Michael Berry

USA

Joseph Bertino

USA

Francois Bertucci

France

Marta Betti

Italy

Vishakha Bhave

USA

Jaishree Bhosle

United Kingdom

Roberto Bianco

Italy

Frederic Bibeau

France

Francois-Clement Bidard

France

Ettore Bidoli

Italy

Giuseppe Bifulco

Italy

Michael Bilous

Australia

Mariano Bizzarri

Italy

Marc Bjurlin

USA

Carmen Blanco-Aparicio

Spain

Giovanni Blandino

Italy

Boris Blechacz

USA

Fonnet Bleeker

Netherlands

Wolfgang Boehmerle

Germany

Johannes Bogaards

Netherlands

David Boone

USA

Kerstin Borgmann

Germany

Josep Borras

Spain

Cristina Bosetti

Italy

Celine Bossard

France

Nadine Bossard

France

Jacoline Bouvy

Netherlands

Dana Bovbjerg

USA

Kjetil Boye

Norway

Allison Boyes

Australia

Paige Bracci

USA

Etienne Brain

France

Elisabeth Brambilla

France

Vivien Bramwell

Canada

Giovanni Brandi

Italy

Burkhard Brandt

Germany

Hiltrud Brauch

Germany

Andreas Brauninger

Germany

Freddie Bray

France

Catherine Brenner

France

Darren Brenner

France

John Bridgewater

United Kingdom

Susanne Briest

Germany

Fadi Brimo

Canada

Ricardo Bringas

Cuba

Jacqueline Bromberg

USA

Christine Brostjan

Austria

Linda Brown

USA

Martin Brown

USA

Melissa Brown

Australia

Richard Brown

USA

Alejandra Bruna

United Kingdom

Andreas Brunner

Austria

Stefania Bruno

Italy

Melinda Btusch Kovacic

USA

Lukas Bubendorf

Switzerland

Malte Buchholz

Germany

Donald Buchsbaum

USA

Kai Budischewski

Germany

Jens Buentzel

Germany

Horst Buerger

Germany

Abdelbaset Buhmeida

Saudi Arabia

Marrije Buist

Netherlands

Ronald Bukowski

USA

Etmar Bulk

Germany

Laki Buluwela

United Kingdom

Harvey Bumpers

USA

Maximilian Burger

Germany

Benedetta Bussolati

Italy

Ralph Buttyan

Canada

Jennifer Byrne

Australia

M.Paz Canadas

Spain

Gabriella Cadoni

Italy

Solange Cadore

Brazil

Sanjun Cai

China

Trinidad Caldes

Spain

Jörg Cammenga

Sweden

Martin Canis

Germany

Christine Canman

USA

David Cano

Spain

Liang Cao

USA

Sumei Cao

China

Xuefang Cao

USA

Gabriel Capella

Spain

Franco Capozza

USA

Vera Cappelletti

Italy

Izilda Cardinalli

British Indian Ocean Territory

Robert Carlisle

United Kingdom

Vinicio Carloni

Italy

De Bolós Pi Carme

Spain

Norman Carr

United Kingdom

Laura Carrassa

Italy

Alfredo Carrato

Spain

Christopher Carroll

United Kingdom

Pierre-François Cartron

France

Giovanni Carulli

Italy

Beatriz Carvalho

Netherlands

Silvia Carvalho

Portugal

Claudio Casali

Italy

Anna Casamichele

Italy

Lynne Cassimeris

USA

Giuliana Cassinelli

Italy

Eduardo Castañeda-Saucedo

Mexico

Alejandra Castanon

United Kingdom

Isabella Castellano

Italy

Adela Castillejo

Spain

Gabriella Castoria

Italy

Felipe A Castro

USA

Patrick Caswell

United Kingdom

Janine Cataldo

USA

Carlos Caulin

USA

Guido Cavaletti

Italy

Erika Cecchin

Italy

Paolo Ceppi

Italy

Güralp Onur Ceyhan

Germany

In Ho Cha

Korea, South

Anees Chagpar

USA

Stephen Chan

Hong Kong

Dhyan Chandra

USA

Urmila Chandran

USA

Alex Chang

Singapore

Tsu-Yi Chao

Taiwan

Nicolas Chapuis

France

Frederic Charron

Canada

Amit Chattopadhyay

USA

Ian Chau

United Kingdom

Jaideep Chaudhary

USA

Satish Cheepala

USA

Hua-Chien Chen

Taiwan

Jing Chen

USA

Miin-Fu Chen

Taiwan

Ming Chen

China

Mukuan Chen

Taiwan

Qingrong Chen

USA

Qing-Xi Chen

China

Shiuan Chen

USA

Wantao Chen

China

Yi-Hau Chen

Taiwan

Jason Chia-Hsien Cheng

Taiwan

Chun-Pin Chiang

Taiwan

John Chiang

USA

Anna Maria Chiaravalli

Italy

Mitsuru Chiba

Japan

Chun-Ru Chien

Taiwan

Yoshitomo Chihara

Japan

Hiroki Chikumi

Japan

Sreenivasulu Chintala

USA

Shih-Hwa Chiou

Taiwan

Rowan Chlebowski

USA

Hervé Chneiweiss

France

Jieun Choi

Korea, South

Matthias Choschzick

Germany

Dipti Chourasia

Malaysia

Edward Chow

Singapore

Thoralf Christoffersen

Norway

Christopher Christophi

Australia

Wei Chua

Australia

Shu-Chun Chuang

United Kingdom

Chen Chun

China

Kwang Chul Chung

Korea, South

Mine Cicek

USA

Artur Cieslar-Pobuda

Sweden

Diana Cittelly

USA

Stephen Clarke

Australia

James Cleary

USA

Nicholas Clement

United Kingdom

Nicholas Clemons

Australia

Martin Clynes

Ireland

Lucio Cocco

Italy

Pierluigi Cocco

Italy

Dawn Cochrane

Canada

Kim Cocks

United Kingdom

Malka Cohen-Armon

Israel

Helen Coleman

United Kingdom

William Coleman

USA

Gisele Colleoni

Brazil

Ben Colleypriest

United Kingdom

Elaina Collie-Duguid

United Kingdom

Barbara Collins

Canada

Brian Collins

USA

Eric Collisson

USA

Giuseppe Colloca

USA

Alessandro Comandone

Italy

Harry Comber

Ireland

Pablo Conesa-Zamora

Spain

Gregory Connolly

USA

Joseph Connor

USA

Matthew Conron

Australia

Patricia Coogan

Uzbekistan

Rebecca Cook

USA

Timothy Cooksley

United Kingdom

Deirdre Coombe

Australia

Scott Coonrod

USA

Timothy Cooper

USA

Valeria Coppola

Italy

Davide Cora'

Italy

Eva Corey

USA

Simona Corso

Italy

Alejandro Corvalan

Chile

David Cosgrove

United Kingdom

Jose Luis Costa

Portugal

Massimo Costantini

Italy

Richard Cote

USA

Claudia Coutinho-Camillo

Brazil

Mark Cowley

Australia

Angela Cox

United Kingdom

Doug Coyle

Canada

Holger Cramer

Germany

Ian Cree

United Kingdom

Chiara Cremolini

Italy

Jo Cresswell

United Kingdom

Ursula Creutzig

Germany

Lucio Crinò

Italy

Alfonso Cristaudo

Italy

Laurent Cronier

France

Amanda Cross

USA

Sidney Croul

Canada

David Crowe

USA

Pamela Crowell

USA

Ana Cuenda

Spain

Zheng Cui

USA

Bryan Cullen

USA

Ruth Cunningham

New Zealand

Artur Czekierdowski

Poland

Rakefet Czerninski

Israel

Tienhan Sandrine Dabakuyo

France

Philipp Dahm

USA

Maria Dalamaga

Greece

Giovanna Damia

Italy

Dragan Damianovich

New Zealand

Reinhard Dammann

Germany

Michael Danilenko

Israel

Philippa Darbre

United Kingdom

Timothy Daskivich

USA

Hiroshi Date

Japan

Ben Davidson

Norway

Michael Davies

USA

Wade Davis

USA

Francesca De Amicis

Italy

Geertruida H. De Bock

Netherlands

Elza De Bruin

United Kingdom

Sophie De Carné

France

Emma De Feo

Italy

Diederick De Jong

United Kingdom

Steven De Jong

Netherlands

Antonella De Luca

Italy

Marc De Perrot

Canada

Aurelien De Reynies

France

Eduardo De Stefani

Uruguay

Chiara De Waure

Italy

Domenico Delia

Italy

Graham Dellaire

Canada

Cyrille Delpierre

France

Melanie Demers

USA

Semra Demokan

Turkey

Gary Deng

USA

Christophe Denoyelle

France

Wu Depei

China

Melissa Derycke

USA

Christèle Desbois-Mouthon

France

Vanessa Deschoolmeester

Belgium

Anjali Deshpande

USA

Omer Devaja

United Kingdom

Gerald Devins

Canada

Neesha Dhani

Canada

Frederic Di Fiore

France

Massimo Di Maio

Italy

Luca Di Tommaso

Italy

Elena Diaz

USA

Ramon Diaz-Uriarte

Spain

Adam Dicker

USA

James Dickinson

Canada

Brenda Diergaarde

USA

Goberdhan Dimri

USA

Wei-Qun Ding

USA

Yu Ding

USA

Andreas Dinkel

Germany

Albena Dinkova-Kostova

United Kingdom

Uta Dirksen

Germany

Juergen Dittmer

Germany

Francesco Dituri

Italy

Hongdo Do

Australia

Katalin Dobra

Sweden

Corinne Doll

Canada

Maria Gabriella Dona'

Italy

Jia-Hong Dong

China

Lanfeng Dong

Australia

Jules Dore

Canada

Thilo Dork

Germany

Olivier Dormond

Switzerland

Valérian Dormoy

USA

Tiffany Doucette

USA

Dinesh Doval

India

Koen Dreijerink

Netherlands

Caroline Drugan

United Kingdom

Catherine Drummond

Sweden

Xiang Du

China

Stan Du Manoir

France

Michel Ducreux

France

Dan Duda

USA

Arkadiusz Dudek

USA

Alfonso Duenas-Gonzalez

Mexico

Hugues Duffau

France

David Duffy

Australia

Stephen Duffy

United Kingdom

Anita Dunbier

New Zealand

Beverly Duncan

USA

Barbara Dunn

USA

Peter Dunscombe

Canada

Ken Dutton-Regester

Australia

Michal Dvorak

Czech Republic

Michael Dyer

USA

Lars Dyrskjot

Denmark

Hans Edmund Eckel

Austria

Konstantinos Economopoulos

USA

Tim Eisen

United Kingdom

Paul Ekert

Australia

Manal El Behery

Egypt

Lina Eliasson

United Kingdom

Elena Elkin

USA

Marwan El-Sabban

Lebanon

Ehab Elzayat

Canada

Jean-Francois Emile

France

Eric Engels

USA

Steven Enkemann

USA

Meira Epplein

USA

Saffet Erdogan

Turkey

Christer Ericsson

Sweden

Hayriye Erkizan

USA

Matthias Ernst

Australia

Silvia Ess

Switzerland

Marie-Louise Essink-Bot

Netherlands

Purificacion Estevez-Garcia

Spain

Tobias Ettl

Germany

Vincenzo Eusebi

Italy

Jeffrey Evans

United Kingdom

Marianne Ewertz

Denmark

Muller Fabbri

USA

Gaetano Facchini

Italy

Stephen Falk

United Kingdom

Weiyi Fang

China

Camile Farah

Australia

Susan Farrington

United Kingdom

Karoly Fatyol

Canada

Adolfo Favaretto

Italy

Elena Favaro

United Kingdom

Peter Fedorocko

Slovakia

Tanja Fehm

Germany

Harriet Feilotter

Canada

Antonio Feliciello

Italy

Jaime Feliu

Spain

Annika Fendler

Germany

Qinghua Feng

USA

Wenli Feng

China

Leandro Fernandez-Perez

Spain

R E Ferner

United Kingdom

Enio Ferreira

Brazil

Irene Ferrer

Spain

Leah Ferrucci

USA

Steven Feske

USA

Stefan Fichtner-Feigl

Germany

Xavier Filella

Spain

Ali Filiz

Turkey

Jorge Filmus

Canada

Eric Finkelstein

Singapore

Emilio Fiore

Italy

Mayer Fishman

USA

Annette Fisseler-Eckhoff

Germany

Liesel Fitzgerald

USA

Chris Flask

USA

Frank Fleischer

Germany

Gudrun Fleischhack

Germany

Michael Flister

USA

Roberto Flores

USA

Marco Folini

Italy

Gunnar Folprecht

Germany

Peter Fong

New Zealand

Nicholas Foreman

USA

Isabelle Fournel

France

Stephen Fox

Australia

Alessandro Franchi

Italy

Alessandra Franzetti Pellanda

Switzerland

Didier Frappaz

France

Antonio Frassoldati

Italy

Milo Frattini

Switzerland

James Freeman

USA

Marie Fridberg

Sweden

Eitan Friedman

Israel

Niels Fristrup

Denmark

Robert Fruscio

Italy

Bryan Fuchs

USA

Ota Fuchs

Czech Republic

Tomoaki Fujioka

Japan

Masashi Fukayama

Japan

Kar-Ming Fung

USA

Yoshiya Furusawa

Japan

Hiroshi Furuse

Japan

Junji Furuse

Japan

Mayuko Furuta

Japan

Alfredo Fusco

Italy

Nozomu Fuse

Japan

Cagatay Gunes

Germany

Jochen Gaedcke

Germany

Bertino Gaetano

Italy

Sanjeev Galande

India

Carlotta Galeone

Italy

Margherita Gallicchio

Italy

Gary Edward Gallick

USA

Brenda Gallie

Canada

Christine Galustian

United Kingdom

Adelina Gama

Portugal

Paolo Gandellini

Italy

Sonal Gandhi

Canada

Olivier Gandrillon

France

Oliver Ganslandt

Germany

Jian-Xin Gao

China

Jing Gao

China

Xian-Shu Gao

China

Juan F Garcia

Spain

Thomas Gardner

USA

Manuela Gariboldi

Italy

Cathie Garnis

Canada

Laura Gatti

Italy

Jean Gaudart

France

Luke Gaughan

United Kingdom

Beatrice Gaugler

France

Karen Geboes

Belgium

Huimin Geng

USA

Jeanine Genkinger

USA

Vassilis Georgoulias

Greece

Felipe Geyer

Brazil

Michelle Ghert

Canada

Kanjaksha Ghosh

India

Sriparna Ghosh

USA

Athina Giagini

Greece

Francesco Gianfagna

Italy

Efi Giannopoulou

Greece

Bruce Giantonio

USA

Spencer Gibson

Canada

Gerrit Gielen

Germany

Joan Gil

Spain

Miguel Gil Gil

Spain

Sebastien Gilbert

Canada

Anthony Gill

Australia

Antonio Z Gimeno García

Spain

Gary Ginsberg

Israel

Ophira Ginsburg

Canada

Daniel Gioeli

USA

Massimo Gion

Italy

Daniela Giorgi

Italy

Ioannis Gioulbasanis

Greece

Elisa Giovannetti

Italy

Mirella Giovarelli

Italy

Dario Giuffrida

Italy

Olivier Glehen

France

Stefania Gobessi

Italy

Jakub Godlewski

USA

Jj Going

United Kingdom

James Goldenring

USA

Kelly Goldsmith

USA

Russell Gollard

USA

Susanne Gollin

USA

Asieh Golozar

USA

Henry Gomez

Peru

Aihua Gong

China

Xue Gong

China

Zhaohui Gong

China

Ivan Gorlov

USA

Kylie Louise Gorringe

Australia

Anita Gothelf

Denmark

Noriko Gotoh

Japan

Martin Götte

Germany

Daria Gotti

Italy

Antonia Gounaris

Greece

Charlie Gourley

United Kingdom

Olga Gra

Russian Federation

Martin Granados

Mexico

Oliver Grauer

Germany

Joel Greenberger

USA

Ewen Griffiths

United Kingdom

Jacques Grill

France

Robert Grimer

United Kingdom

Christoph Grimm

Austria

Chloe Grimmett

United Kingdom

Peter Grimminger

USA

Tom Kristian Grimsrud

Norway

Patricia Groenen

Netherlands

Nicole Grosse

Switzerland

Yueqing Gu

China

Fiorella Guadagni

Italy

Maria Rosaria Gualano

Italy

Xiaoxiang Guan

China

Xin-Yuan Guan

Hong Kong

Monika Gube

Germany

Matthias Guckenberger

Germany

Kishore Guda

USA

Mickael Guedj

France

Corina Guethlin

Germany

Gulnur Guler

Turkey

William Gullick

United Kingdom

Alexander Guminski

United Kingdom

Mehmet Gunduz

Japan

Lin Guo

China

Xiang Guo

USA

Digant Gupta

USA

John Haanen

Netherlands

Nikolas Haass

Australia

Christina Hackl

Germany

Michael Haddock

USA

Andreas Hadjisavvas

Cyprus

Jeffrey Hager

USA

Caj Haglund

Finland

Samer Hakim

Germany

Michiyuki Hakozaki

Japan

Timo Hakulinen

Finland

John Haley

USA

Janet Hall

France

Paul Hamel

Canada

Omid Hamid

USA

Catherine (Hye-Won) Han

New Zealand

Torben Hansen

Denmark

Chunhai Hao

USA

Kristina Hardy

USA

William Harris

USA

Mary Hart

USA

Mikael Hartman

Singapore

Sylvia Hartmann

Germany

Susanne Hartwig

France

Catherine Harwood

United Kingdom

Sandra Z Haslam

USA

Cesrae Hassan

Italy

Jens Hasskarl

Germany

Anna Hawkes

Australia

Eliza Hawkes

United Kingdom

Valtteri Häyry

Finland

Aiwu He

USA

Biao He

USA

Dongmei He

China

Qiyang He

China

John Healey

USA

Mark Heaney

USA

Shantel Hébert-Magee

USA

Rakesh Heer

United Kingdom

Petra Heffeter

Austria

Eva Hellmén

Sweden

Alain Hendlisz

Belgium

Daniel Heng

USA

Brittney-Shea Herbert

USA

Kerstin Hermelink

Germany

Ingrid Herr

Germany

Peter Hersey

Australia

William Heymann

USA

Heidegard Hilbig

Germany

Elizabeth Hill

USA

Christine Hill-Kayser

USA

Sebastian Hinde

United Kingdom

Shiro Hinotsu

Japan

Yoshihiko Hirohashi

Japan

Takashi Hirose

Japan

Joerg Hoehfeld

Germany

Robert Hoffman

USA

Wolfgang Hoffmann

Germany

Craig Hofmeister

USA

Veronica Höiom

Sweden

Stefan Holdenrieder

Germany

Hubert Hondermarck

Australia

Peter Horak

Austria

Craig Horbinski

USA

Yuichi Hori

Japan

Jason Hornick

USA

Michael Horsman

Denmark

David Horst

Germany

Peng Hou

China

Rémi Houlgatte

France

Carrie House

USA

Christopher Hovens

Australia

Doris Howell

Canada

Edmond Hsieh

Taiwan

Jian-Kun Hu

China

Lifu Hu

Sweden

Longqin Hu

USA

Zhiwei Hu

USA

Zheng Hua-Chuan

China

Annie Huang

Canada

Cai Huang

USA

Cheng Huang

USA

Min Huang

USA

Shile Huang

USA

Shiu-Feng Huang

Taiwan

Sidong Huang

Netherlands

Wenlin Huang

China

Xinsheng Huang

China

Richard Hubbard

United Kingdom

Christian Hübbers

Germany

Peter Huber

Gibraltar

Samuel Huber

Germany

Amanda Hummon

USA

Darel Hunting

Canada

Fazlul Huq

Australia

Sara Hurvitz

USA

Damian Hussey

Australia

Evelyn Hutt

USA

Chung Feng Hwang

Taiwan

Ezzeldin Ibrahim

Saudi Arabia

Ramazan Idilman

Turkey

Thomas Iftner

Germany

Peter Igaz

Hungary

Stephen Ihrler

Germany

Naoki Ikenaga

USA

Dora Il'Yasova

USA

S Ashraf Imam

USA

Haruhiko Imamoto

Japan

Mutsumi Inaba

Japan

Mikito Inokuchi

Japan

Peter Inskip

USA

Marilena Iorio

Italy

Vincenzo Ippolito

Italy

Joseph Irgon

USA

William Isaacs

USA

Mitsuru Ishizuka

Japan

Farhad Islami

France

Jean-Paul Issartel

France

Hiroaki Itamochi

Japan

Yoshinori Itoh

Japan

Nina Iversen

Norway

Yasuo Iwadate

Japan

Jakob Izbicki

Germany

Mike Jackson

United Kingdom

Francis Jacob

Switzerland

Bart Jacobs

Belgium

Judith S Jacobson

USA

Norman Jaffe

USA

A Jager

Netherlands

Rolf Jaggi

Switzerland

Pawel Jagodzyki

Poland

Rakesh Jain

USA

Vladimir Jakovljevic

Serbia

Nigel Jamieson

United Kingdom

Nina Jancar

Slovenia

Filip Janku

USA

Frank Jansman

Netherlands

Emiel Janssen

Norway

Marie-Claude Jaurand

France

Luis F. Jave-Suarez

Germany

Tille Jc

Switzerland

Aaron Jeffs

New Zealand

Jaroslav Jelinek

USA

Ahmedin Jemal

USA

Yung-Ming Jeng

Taiwan

Brendan Jenkins

Australia

Valerie Jenkins

United Kingdom

Claire Jenkinson

United Kingdom

Randy Jensen

USA

Sang Ryong Jeon

Korea, South

Carmen Jerónimo

Portugal

Hongbin Ji

China

Bing-Hua Jiang

USA

Chenggang Jiang

China

Kai Jiang

China

Xuejuan Jiang

USA

Chen Jiawei

China

Bilian Jin

USA

Markus Joerger

Switzerland

Tom John

Australia

Lars Jordheim

France

Anthony Joshua

Canada

Chaeyong Jung

Korea, South

Young Jung

Korea, South

Christina Justenhoven

Germany

R K

United Kingdom

Geoffrey Kabat

USA

Martin Kaelin

Switzerland

Christoph Kahlert

Germany

Shahla Kakoei

Iran

Galatea Kallergi

Greece

Tuula Kallunki

Denmark

Konstantine Kalogeras

Greece

Gentian Kaloshi

Albania

Masahiro Kami

Japan

Mustapha Kandouz

USA

Eleanor Kane

United Kingdom

Jung Hun Kang

Korea, South

Hiroaki Kanouchi

Japan

Chryso Kanthou

United Kingdom

Ryu Kanzaki

Japan

Kuo-Jang Kao

Taiwan

Dev Karan

USA

Henning Karlsson

Sweden

Judith Karp

USA

Peter Karran

United Kingdom

Sabine Kasimir-Bauer

Germany

Venkat Katkoori

USA

Noriyuki Katsumata

Japan

Matthew Katz

USA

Marja Kaunonen

Finland

Preet Kaur

India

Toshiaki Kawai

Japan

Kazuyuki Kawakami

Japan

Kathleen Melissa Ke

United Kingdom

Bhumsuk Keam

Korea, South

Dorothy Keefe

Australia

Daniel Keizman

Israel

Malcolm Kell

Ireland

Ruth Keri

USA

Keith Kerr

United Kingdom

David Kessel

USA

Marco Kesting

Germany

Khandan Keyomarsi

USA

Gazala Khan

USA

Abdel-Majid Khatib

France

Hung Khong

USA

Ui Soon Khoo

China

La Creis Kidd

USA

Ergin Kilic

Germany

Saadettin Kilickap

Turkey

Eugene Kim

USA

Isaac Kim

USA

Jin Kim

Korea, South

Jung Gu Kim

Korea, South

Sang-We Kim

Korea, South

Seung-Ki Kim

Korea, South

Soo-Youl Kim

Korea, South

Yeul Hong Kim

Korea, South

Yong Sung Kim

Korea, South

Yong-June Kim

Korea, South

Yoon Jun Kim

Korea, South

Younghak Kim

Japan

You-Sun Kim

Korea, South

Yu Ri Kim

Korea, South

Cari Kitahara

USA

Joji Kitayama

Japan

Henry Kitchener

United Kingdom

Christopher Klebanoff

USA

Sonja Klebe

Australia

Robert Klein

USA

Pamela Klingbeil

United Kingdom

Stian Knappskog

Norway

Sebastian Kobold

Germany

Moritz Koch

Germany

Joerg Koenig

Germany

Eng-Siew Koh

Australia

Fortune Kohen

Israel

Junkichi Koinuma

Japan

Santhi Konduri

USA

Ja Seung Koo

Korea, South

Larissa Korde

USA

Hasan Korkaya

USA

Kostas Kostarelos

United Kingdom

Aleksandar Kostic

USA

Athanasios Kotsakis

Greece

Panteleimon Kountourakis

Cyprus

Mariusz P. Kowalewski

Switzerland

Oliver Krämer

Germany

Robert Kratzke

USA

Steffen Krause

Austria

Marie-Adele Kress

USA

Steven Kridel

USA

Glen Kristiansen

Germany

Niels Kroman

Denmark

Patricia Kruk

USA

Geoffrey Ku

USA

Jian Kuang

USA

Dieter Kube

Germany

Gregory Kucera

USA

Rajiv Kumar

Germany

Sanath Kumar

USA

Sherko Kümmel

Germany

Hiroki Kuniyasu

Japan

Hsing-Chin Kuo

Taiwan

Yasuhiro Kuramitsu

Japan

Ryoji Kushima

Japan

Sergei Kusmartsev

USA

Shigeru Kusumoto

Japan

Hiroko Kuwabara

Japan

Alastair Kyle

Canada

Maria Kyrgiou

United Kingdom

Anastasios Kyriazoglou

Greece

Caterina La Porta

Italy

Carlo La Vecchia

Italy

Manuela Lacerda

Portugal

Herwig Lackner

Austria

Florian Laenger

Germany

Chann Lagadec

USA

George Laking

New Zealand

Brenda Laky

Austria

Wendy Wing Tak Lam

Hong Kong

Rebecca Lamb

United Kingdom

Aude Lamy

France

Claudia Lanari

Argentina

Rachelle Lanciano

USA

Danny Landau

USA

Matteo Landriscina

Italy

Marene Landström

Sweden

Sven Lang

Germany

Simon Langdon

United Kingdom

John Lange

USA

Primo Lara

USA

Annette K. Larsen

France

Inger Kristin Larsen

Norway

Timothy Lash

USA

Igor Latorzeff

France

Harold Lau

Canada

Loretta Lau

Australia

Elizabeth Lawlor

USA

Kate Lawlor

Australia

Ben Lawrence

USA

Andreas Lazaris

Greece

Matthew Lazzara

USA

John Le Quesne

United Kingdom

Romuald Le Scodan

France

Dermot Leahy

Ireland

Jenny Leary

Australia

Lilian Lechner

Netherlands

Matthias Lechner

United Kingdom

Thierry Lecomte

France

Han Chu Lee

Korea, South

Hsin-Chen Lee

Taiwan

Kin Chung Lee

Hong Kong

Kyoung Mu Lee

Korea, South

Peter Peng Foo Lee

Singapore

Randy Legerski

USA

Mario Leitao Jr

USA

Andreas Leithner

Austria

Wolfgang Leitner

USA

Marcis Leja

Latvia

Manuel Lemos

Portugal

Nikoletta Lendvai

USA

Dido Lenze

Germany

Leondios Leondiadis

Greece

Wey Leong

Canada

Laurent Lessard

USA

Fabienne Lesueur

France

Yu-Wei Leu

Taiwan

Elaine Leung

United Kingdom

Miroslav Levy

Czech Republic

Guojun Li

USA

Jian Li

Denmark

Ji-Liang Li

United Kingdom

Xiao-Nan Li

USA

Bertrand Liagre

France

Theodore Liakakos

Greece

Kan Liao

China

Zhongxing Liao

USA

Cornelia Liedtke

Germany

Nancy Lill

USA

Megan Lim

USA

Min Kyung Lim

Korea, South

Soo Jeong Lim

Korea, South

Unhee Lim

USA

Wan-Teck Lim

Singapore

Stefan Limmer

Germany

Janusz Limon

Poland

Patrizia Limonta

Italy

Temduang Limpaiboon

Thailand

Che Lin

Taiwan

Chi Lin

USA

Chin-Tarng Lin

Taiwan

Chunche Lin

Taiwan

Jin-Ching Lin

Taiwan

Kenneth Lin

USA

Steven Lin

USA

Corinne Linardic

USA

Mark Linch

United Kingdom

Annika Lindblom

Sweden

Sara Lindstrom

USA

Lorraine Lipscombe

Canada

Lara Lipton

Australia

Nikolai Lisitsyn

Russian Federation

Bingya Liu

China

Fu-Tong Liu

USA

Jinyi Liu

China

Ren-Shyan Liu

Taiwan

Shuzhen Liu

Canada

Tianshu Liu

China

Yang Liu

USA

Yueyong Liu

USA

Yunpeng Liu

China

Zhihui Liu

USA

Marcela Lizano

Mexico

Shane Lloyd

USA

Lorenzo Lo Muzio

Italy

Susan Logan

USA

Tatiana Loganovskaja

Ukraine

Anna Lokshin

USA

Lai Meng Looi

Malaysia

Herbert Loong

Hong Kong

Gilberto De Lima Lopes

Singapore

María Luisa López

Spain

Antonio Lopez-Beltran

Spain

Jose Antonio Lopez-Guerrero

Spain

Armando López-Guillermo

Spain

Gloria Lopez-Mckee

USA

Marek Los

Germany

Wenhui Lou

China

Bo Lu

USA

Yi Lu

China

Margreet Lüchtenborg

United Kingdom

Giovanni Lughezzani

Italy

Limin Lun

China

Hong Lok Lung

Hong Kong

Gary Lyman

USA

Heiko Muhl

Germany

Hongxia Ma

China

Huiyan Ma

USA

Patrick Ma

USA

Shenglin Ma

China

Ruth Mackinnon

Australia

Una Macleod

United Kingdom

Clelia Madeddu

Italy

Srinivasan Madhusudan

United Kingdom

Hiroshi Maeda

Japan

Dianna Magliano

Australia

Sven Mahner

Germany

Georges Maire

Canada

Haroon Majeed

United Kingdom

Dragomira Majhen

Croatia

Ireneusz Majsterek

Poland

Markus Mäkinen

Finland

Liza Makowski

USA

Nik Makretsov

Canada

Roberta Malaguarnera

Italy

Núria Malats

Spain

Stefano Malerba

Italy

Bernard Malfoy

France

David Malkin

Canada

Maria Cristina Manara

Italy

Mahitosh Mandal

India

Tarun Mandal

USA

Lingegowda Mangala

USA

Ferdinando Mannello

Italy

Arto Mannermaa

Finland

Elisabetta Marangoni

France

Raffaella Marcheselli

Italy

Sergio Marchini

Italy

Caterina Marchiò

Italy

Francesca Marini

Italy

Maria Marino

Italy

Angela Mariotto

USA

Udo R. Markert

Germany

Nicholas Marko

United Kingdom

Athina Markou

Greece

Iván Márquez-Rodas

Spain

Guido Martignoni

Italy

Finian Martin

Ireland

Juan Martinez

Spain

Ramon Martinez

Germany

Victor Martinez

Canada

Eva Martinez-Balibrea

Spain

Luis Martinez-Lostao

Spain

Jorge Martin-Perez

Spain

Jose Ignacio Martin-Subero

Spain

Mario Mascalchi

Italy

Gianluca Masi

Italy

Anne Mason

United Kingdom

Daniela Matei

USA

Xavier Matias-Guiu

Spain

Koichi Matsuda

Japan

Koji Matsumoto

Japan

Keitaro Matsuo

Japan

Junko Matsuzaki

USA

Jaime Matta

Puerto Rico

Harald Matthes

Germany

Giuseppe Matullo

Italy

Edmundo Mauad

Brazil

Loredana Mauro

Italy

Christine Mauz-Körholz

Germany

Christian Mawrin

Germany

Elisa May

Germany

Christine Mayr

USA

Marie-Helene Mayrand

Canada

Frédéric Mazurier

France

Brooke Mccartney

USA

David Mccormick

USA

Michael Mcdevitt

USA

Michael Mcgee

USA

Mark Mckeage

New Zealand

Ray Mclaughlin

Ireland

Donald Mcmillan

United Kingdom

Cristina Mecucci

Italy

Rui Medeiros

Portugal

Alfons Meindl

Germany

Sreelatha Meleth

USA

Lene Mellemkjaer

Denmark

Apostolos Menegakis

Germany

Xiangbing Meng

USA

Rolf Mentlein

Germany

Neil Merrett

Australia

Janet Mertz

USA

Stefania Meschini

Italy

Eric Metzen

Germany

Jens E. Meyer

Germany

Christoph Michalski

Germany

Rigaud Michel

France

Carine Michiels

Belgium

Antimo Migliaccio

Italy

Lucia Migliore

Italy

Michele Davide Mignogna

Italy

Yasuhiro Miki

Japan

Linda Mileshkin

Australia

Hrvoje Miletic

Norway

Imari Mimura

Japan

Hiroyuki Mineta

Japan

Olivier Mir

France

Lisa Mirabello

USA

Leonardo Mirandola

Italy

Elizabeth Mitchell

United Kingdom

Rama Mittal

India

Koh Miura

Japan

Etsuko Miyagi

Japan

Makito Miyake

Japan

Yasuyoshi Miyata

Japan

Motoki Miyazawa

Japan

Dominik Modest

Germany

Markus Moehler

Germany

Rabab Ahmed Mohammed

Egypt

Anant Mohan

India

Samuel Mok

USA

Kefah Mokbel

United Kingdom

Sonia Molina-Pinelo

Spain

Francesca Molinari

Switzerland

Ute Moll

USA

Stefan Paul Mönig

Germany

Liliana Montella

Italy

Ronald Moore

Canada

Victor Moreno

Spain

Miriam Morey

USA

Meredith Morgan

USA

Yoichiro Moriya

Japan

Masatsugu Moriyama

Japan

Luc Morris

USA

David Morrison

United Kingdom

Fernanda B. Morrone

Brazil

Sean Mortimore

United Kingdom

Sergei Moshkovskii

Russian Federation

Dick Mosser

Canada

Marcella Mottolese

Italy

Giannis Mountzios

France

Seyed Mohsen Mousavi

Germany

Berit Maria Mueller

Germany

Wolfgang Mueller-Klieser

Germany

Ken-Ichi Mukaisho

Japan

Thomas Muley

Germany

A.K Munirajan

India

Cristina Munoz-Pinedo

Spain

Jordi Muntané

Spain

Kei Muro

Japan

Ola Myklebost

Norway

Keun Na

Korea, South

Kazuki Nabeshima

Japan

Ghulam Nabi

United Kingdom

Ulrich Nachbur

Australia

Jose Nacher

Japan

Mark Nachtigal

Canada

Ali Naderi

USA

Nagalakshmi Nadiminty

USA

Raghuram Nagarathna

India

Kazunori Nagasaka

Japan

Eduardo Nagore

Spain

Iris Nagtegaal

Netherlands

Gyorgy Nagy

Hungary

John Nagy

USA

Zeina Nahleh

USA

Pradeep Naik

India

Jose Najera

USA

Atsushi Nakajima

Japan

Takahiro Nakajima

Japan

Masayoshi Nakanishi

Japan

Kohei Nakata

Japan

Tetsuya Nakatsura

Japan

Tomio Nakayama

Japan

Joo-Hyun Nam

Korea, South

Pratima Nangia-Makker

USA

Margherita Nannini

Italy

Yoshio Naomoto

Japan

Steven Narod

Canada

Najmunnisa Nasreen

USA

Cord Naujokat

Germany

Wojciech Naumnik

Poland

Ruta Navakauskiene

Lithuania

Shaunak Navalkissoor

United Kingdom

Nora Navone

USA

Roman Nawroth

Germany

Richard Neal

United Kingdom

Len Neckers

USA

Kari Nejak

USA

Valentina Nekljudova

Germany

Brad Nelson

Canada

Heather Nelson

USA

Gila Neta

USA

Kristi Neufeld

USA

Jiri Neuzil

Australia

Paolo Neviani

USA

Robert Newton

Australia

David Ng

USA

Simon Ng

Hong Kong

Wei Cheong Ngeow

Malaysia

Nghi Nguyen

USA

Edward Nickoloff

USA

Alcina Nicol

Brazil

Giordano Nicoletti

Italy

Morten Schak Nielsen

Denmark

Akira Nifuji

Japan

Jonas Nilsson

Sweden

Hiroshi Nishida

Japan

Naoshi Nishida

Japan

Hiroyoshi Nishikawa

Japan

Reiki Nishimura

Japan

Benjamin Nisman

Israel

Anna Niwinska

Poland

Maximilian Niyazi

Germany

Rosa Noguera

Spain

Sung Hoon Noh

Korea, South

Daniel Normolle

USA

Francesco Novelli

Italy

Rajesh Kumar Nv

USA

Fiemu Nwariaku

USA

Koji Oba

Japan

Takumi Ochiai

Japan

Katsutoshi Oda

Japan

Donal O'Donoghue

United Kingdom

Norma O'Donovan

Ireland

Ina Oehme

Germany

Michael Oertel

USA

Elizabeth O'Flynn

United Kingdom

Kalu Ogbureke

USA

Olorunseun Ogunwobi

USA

Daniel Oh

USA

Rika Ohkuma

USA

Seiichi Okabe

Japan

Hideho Okada

USA

Morihito Okada

Japan

Alicia Okines

United Kingdom

Hester Oldenburg

Netherlands

Magali Olivier

France

David Olmos

Spain

Abdulfatai Olokoba

Nigeria

Catherine Olsen

Australia

Geraldine O'Neill

Australia

Kim O'Neill

USA

Kong Wee Ong

Singapore

Mykola Onyshchenko

USA

Akishi Ooi

Japan

Kirstie Opstad

United Kingdom

Eileen O'Reilly

USA

Michael O'Rorke

United Kingdom

Shinji Osada

Japan

Mitsuhiko Osaki

Japan

Kayo Osawa

Japan

Irina Ostrovnaya

USA

Eleanor O'Sullivan

Ireland

Sandra Otoole

Australia

Laura Ottini

Italy

Oliver Ottmann

Germany

Ouathek Ouerfelli

USA

Annelies Overbeek

Netherlands

Atsushi Ozawa

Japan

Isao Oze

Japan

Sevket Ozkaya

Turkey

Jin Chul Paeng

Korea, South

Jens Pahl

Netherlands

Joel Palefsky

USA

Claudia Palena

USA

Athanasios Pallis

Greece

Johan Pallud

France

Johannes Pammer

Austria

Georgios Pampalakis

Greece

Hongming Pan

China

Massimo Pancione

Italy

Giuseppe Pandini

Italy

Christos Paraskeva

United Kingdom

Olivier Pardo

United Kingdom

Joana Paredes

Portugal

Marie-Elise Parent

Canada

François Paris

France

Do Youn Park

Korea, South

Hee Chul Park

Korea, South

Jae Yong Park

Korea, South

Jong Kuk Park

Korea, South

Joo-Cheol Park

Korea, South

Margaret Park

USA

Sohee Park

Korea, South

Young Park

Korea, South

Young Joo Park

Korea, South

Janet Parsons

Canada

Boris Pasche

USA

Fabio Pastorino

Italy

Ugo Pastorino

Italy

Kho Patricia

Australia

Hyland Paula

USA

Nicholas Pavlidis

Greece

Geoffrey Payne

United Kingdom

Stuart James Peacock

Canada

Paolo Pedrazzoli

Italy

Paivi Peltomaki

Finland

Bi-Wen Peng

China

Ling Peng

France

Fabio Penna

Italy

Marzia Pennati

Italy

Beata Peplonska

Poland

Paola Perego

Italy

Ignacio Perez De Castro

Spain

Eduardo Perez Salazar

Mexico

Patricia Perez-Galan

Spain

Rebecca Perkins

USA

Daniele Perna

United Kingdom

Anna Perri

Italy

Federica Perrone

Italy

Saskia Persoon

Netherlands

Jenny Persson

Sweden

Michelle Perugini

Australia

Augusto Pessina

Italy

Bernhard Pestalozzi

Switzerland

Shajan Peter

USA

Jiri Petera

Czech Republic

Paolo Peterlongo

Italy

Roberto Petrioli

Italy

Matthieu Peyre

France

Shelly Peyton

USA

Markus Pfirrmann

Germany

Giao Phan

USA

Kristin Phillips

USA

Phoebe Phillips

Australia

Alain Piché

Canada

Martin Pichler

Austria

Jean-Yves Pierga

France

Phillip Pierorazio

USA

Christian Pilarsky

Germany

Shari Pilon-Thomas

USA

Kirit Pindolia

USA

António Pinto

Portugal

Luis Pinto

Brazil

Pamela Pinzani

Italy

Vincenzo Pitini

Italy

Roberto Piva

Italy

John Plastaras

USA

Manu Platt

USA

Marta Ewa Polak

United Kingdom

Buelent Polat

Germany

Craig Pollack

USA

Marina Pollan

Spain

Ewa Pomianowska

Niger

Odilia Popanda

Germany

Isabel Poschke

Sweden

Emily Power

United Kingdom

Nicole Pratt

Australia

Timothy Price

Australia

Hetty Prinsen

Netherlands

Kathleen Pritchard

Canada

Valentina Profumo

Italy

Paolo Provenzano

USA

Sandi Pruitt

USA

Ida Pucci-Minafra

Italy

Ramon M Pujol

Spain

Karen Pulford

United Kingdom

Raj K. Puri

USA

Lajos Pusztai

USA

Zheng Qingyou

China

Maria Quarto

Italy

Leticia Quintanilla-Fend

Germany

Harry Quon

USA

Curzio Rã¼Egg

Switzerland

Annette Raabe

Germany

Milan Radovich

USA

Nuh Rahbari

Germany

Km Rahman

USA

Marius Raica

Romania

Eva Rajnavolgyi

Hungary

Janusz Rak

Canada

Steve Ralph

Australia

Giuliano Ramadori

Germany

Bram Ramaekers

Netherlands

Pranela Rameshwar

USA

Theodoros Rampias

Greece

Heribert Ramroth

Germany

Sophia Ran

USA

Keith Rand

Australia

Girolamo Ranieri

Italy

Elisabetta Rapiti

Switzerland

Shahazd Raza

USA

Francisco X. Real

Spain

Michele Redell

USA

Helen Reeves

United Kingdom

Peter Reichardt

Germany

Rui Manuel Reis

Portugal

Cp Ren

China

Huan Ren

China

Oliver Renner

Spain

Elizabeth A. Repasky

USA

Santhoshkumar Retnabai

India

Pascale Reverdiau

France

Andy Reynolds

United Kingdom

Domenico Ribatti

Italy

Karin Ribi

Switzerland

Donald Richards

USA

Jennifer Richer

USA

Susan Richman

United Kingdom

Rachel Riechelmann

Brazil

James Rigas

USA

Brian Rini

USA

Ashley Rivenbark

USA

Laurent Rivory

Australia

Juan Carlos Roa

Chile

Mack Roach

USA

Karin Roberg

Sweden

Caroline Robert

France

Jacques Robert

France

Helen Roberts

New Zealand

Gavin Robertson

USA

Max Robinson

USA

Trude Eid Robsahm

Norway

Marco Bl Rocchi

Italy

Rafael Rocha

Brazil

David Roder

Australia

Ligia Rodrigues

Portugal

Jakub Rohlena

Czech Republic

Sabine Rohrmann

Switzerland

Andreas Roidl

Germany

Jesse Roman

USA

Annette Romanski

Germany

Martin Roosli

Switzerland

Alessandra Rosati

Italy

Calvin Roskelley

Canada

Helen Ross

USA

Lone Ross

Denmark

Paul Ross

United Kingdom

Charles Rosser

USA

Jochen Rossler

Germany

Matthias Rostock

Switzerland

Matteo Rota

Italy

Rossella Rota

Italy

Lawrence Roth

USA

Wilfried Roth

Germany

Nada Rotovnik Kozjek

Slovenia

Gael Roue

Spain

C. Bart Rountree

USA

G.Enrico Rovati

Italy

Partha Roy

USA

Rebecca Roylance

United Kingdom

Luca Roz

Italy

Enrique Rozengurt

USA

Volker Rudat

Saudi Arabia

Montserrat Rue

Spain

Oscar M Rueda

United Kingdom

Thomas Ruhstaller

Switzerland

Alvaro Ruibal

Spain

Christoph Rummel

Germany

Antonio Russo

Italy

Lisa Rydén

Sweden

Aki Ryo

Japan

Jeong-Seon Ryu

Korea, South

Nabil Saba

USA

Motoyasu Sagawa

Japan

Kazutaka Saito

Japan

Núria Sala

Spain

Ramon Salazar

Spain

Ashok Saluja

USA

Emmanuelle Samalin

Ethiopia

Shamith Samarajiwa

United Kingdom

Ricardo Sanchez Prieto

Spain

Patricia Sancho

Spain

Samith Sandadi

USA

Bruno Sangro

Spain

Hiroshi Sano

Japan

Regina Santella

USA

Takaaki Sasaki

Japan

Eiichi Sato

Japan

Christobel Saunders

Australia

Suzana Savkovic

USA

Luca Scapoli

Italy

Chris Scarlett

Australia

Marco Scarpa

Italy

Mario Scartozzi

Italy

Ghislaine Scélo

USA

Michael Schaapveld

Netherlands

Matthew Schabath

USA

Karl-Ludwig Schaefer

Germany

Knut Schäkel

Germany

Karl Schaller

Switzerland

Tobias Schatton

USA

Dorthe Schaue

USA

Kristina Schee

Norway

Silke Schelenz

United Kingdom

William Schelman

USA

Arnaud Scherpereel

France

Aaron Schetter

USA

Michael Scheurer

USA

Steven Schild

USA

Jeffrey Schlom

USA

Fernando Schmitt

Portugal

Manfred Schmitt

Germany

Bryan Schneider

USA

Guenter Schneider

Germany

Patricia Schoenlein

USA

Mario Schootman

USA

Wolfgang Schuette

Germany

Scott Schuetze

USA

Fredrick Schumacher

USA

Christian Schwentner

Germany

Joannah Score

United Kingdom

Eva Segelov

Australia

Christoph Seidel

Germany

Andrew Seidman

USA

Motoharu Seiki

Japan

Volkhard Seitz

Germany

Florin Selaru

USA

Morgan Sellers

USA

Andrei Seluanov

USA

Robert Semrau

Germany

Christine Sers

Germany

Cristiana Sessa

Switzerland

Virginia Seybold

USA

Alex Sgambato

Italy

Keerti Shah

USA

Shohreh Shahabi

USA

Chandrakumar Shanmugam

India

Jenny Shannon

Australia

Huanjie Shao

USA

Shahrokh Shariat

USA

Dr Atul Sharma

India

Tyson Sharp

United Kingdom

Qing-Bai She

USA

Chiung-Chyi Shen

Taiwan

Han-Ming Shen

Singapore

Karen Sherman

USA

Simon Sherman

USA

Kirti Shetty

Vanuatu

Lalita Shevde-Samant

USA

Wenyin Shi

USA

Yongquan Shi

China

Yih-Horng Shiao

USA

Darryl Shibata

USA

Kiyosumi Shibata

Japan

Wen-Ling Shih

Taiwan

Hideaki Shimada

Japan

Masahito Shimizu

Japan

Shoji Shimoyama

Japan

Tsutomu Shimura

Japan

Satoshi Shiono

Japan

Protul Shrikant

USA

Oliver Sieber

Australia

Markus Siegelin

USA

D. Robert Siemens

Canada

Parames Sil

India

Ismael Silva

Brazil

Susana Silva

Portugal

Ida Silvestri

Italy

Rosella Silvestrini

Italy

Dmitri Simberg

USA

Iris Simon

Netherlands

Sara Simonetti

Spain

Amar Singh

USA

Harminder Singh

Canada

Fabrice Sircoulomb

Canada

Chok Siu Ho

Hong Kong

Chris Skedgel

Canada

Ingiridur Skirnisdottir

Sweden

Gillian Smith

United Kingdom

Stuart Smith

United Kingdom

Marcelo Soares

USA

Jonathan Soboloff

USA

Alberto Sobrero

Italy

Tomotaka Sobue

Japan

Isabelle Soerjomataram

Netherlands

Rikke Søgaard

Denmark

Stephanie Sohl

USA

Brigitte Sola

France

Bao Song

China

Kyo Young Song

Korea, South

Amir Sonnenblick

Israel

Makoto Sonobe

Japan

Suneet Sood

Malaysia

Sverre Sörenson

Sweden

Néstor Soria

Argentina

Jamshid Sorouri Khorashad

USA

Giovanni Sotgiu

Italy

José Luis Soto

Spain

David Soto Pantoja

USA

Ioannis Souglakos

Greece

Gursel Remzi Soybir

Turkey

Rainer Spang

Germany

Rebecca Speck

USA

Ernst-Jan Maria Speel

Netherlands

David Spigel

USA

Douglas Spitz

USA

Thilo Sprenger

Germany

Padmini Srinivasan

USA

Krishna Bajee Sriram

Australia

Kshitij Srivastava

USA

Silvia Stacchiotti

Italy

Synnöve Staff

Finland

Stefania Staibano

Italy

Annette Stanton

USA

Julia Starmann

Germany

Sandra Starnes

USA

Martine Stead

United Kingdom

Jason Steel

USA

Renske Steenbergen

Netherlands

Peter Stehle

Germany

Alexander Stein

Germany

Karen Steindorf

Germany

Diana Steinmann

Germany

Sally Stephenson

Australia

Julie Sterling

USA

Elizabeth Stier

USA

Thomas Stinchcombe

USA

Sebastian Stintzing

Germany

Angela Stokes

United Kingdom

Clelia Tiziana Storlazzi

Italy

Christos Stournaras

Greece

Sandra Strauss

United Kingdom

Klaus Strebhardt

Germany

Antonio Strillacci

Italy

Michael Stroehlein

Germany

Changqing Su

China

Ying-Hsiu Su

USA

Eric Suba

USA

Eisaburo Sueoka

Japan

Kazuysohi Suga

Japan

Kenichi Sugihara

Korea, South

Kazushi Sugimoto

Japan

Karijn Suijkerbuijk

Netherlands

Gordana Supic

Serbia

Jordi Surralles

Spain

Norman Sussman

USA

Siobhan Sutcliffe

USA

Leslie Sutherland

Canada

Koichi Suzuki

Japan

Nao Suzuki

Japan

Reiko Suzuki

Japan

Takashi Suzuki

Japan

Randi Syljuåsen

Norway

Fournel-Gigleux Sylvie

France

Vana Sypsa

Greece

Astri Syse

Norway

Cezary Szczylik

Poland

Richard Szydlo

United Kingdom

Andrea 'T Mannetje

New Zealand

Maria Letiza Taddei

Italy

Elda Tagliabue

Italy

Naveen Tahasildar

India

Ming-Hong Tai

Taiwan

Daniel Taillandier

France

Chiaki Takahashi

Japan

Daiya Takai

Japan

Noriyuki Takai

Japan

Tetsuo Takehara

Japan

Keizo Takenaga

Japan

Tomoyoshi Takenaka

Japan

Osamu Takeuchi

Japan

Rulla Tamimi

USA

David Tan

United Kingdom

Eng Huat Tan

Singapore

Lijie Tan

China

Fumiaki Tanaka

Japan

Nobumichi Tanaka

Japan

Takuji Tanaka

Japan

Flavio Tancioni

Italy

Daolin Tang

USA

Andrea Tannapfel

Germany

Jin Tao

USA

Qian Tao

Hong Kong

Coya Tapia

Switzerland

Natasa Tasevska

Macedonia

Rosemary Tate

United Kingdom

Ryosuke Tateishi

Japan

Jeremy Taylor

USA

Matthew J. Taylor

USA

Justin Teissie

France

Julio Teixeira

Brazil

Clemens Tempfer

Germany

Arnoud Templeton

Canada

Yoshito Terai

Japan

Anna Tesei

Italy

Ugo Testa

Italy

Ivan Weng Keong Tham

Singapore

Lai San Tham

Singapore

Armin Thelen

Germany

Achilleas Theocharis

Greece

Sam Thiagalingam

USA

Emilie Thivat

France

Cynthia Thomson

USA

Henrik Thorlacius

Sweden

Carmelo Tibaldi

Italy

Jozsef Timar

Hungary

Virginia Tirino

Italy

Matthew Tirrell

USA

Verena Maria Tischler

Switzerland

Tilman Todenhöfer

Germany

Valentina Todorova

USA

Masakazu Toi

Japan

Adam Toll

USA

M Tomasetti

Italy

Masaki Tomita

Japan

Johanna Tommiska

Finland

Naohisa Tomosugi

Japan

Sarah Tonack

Germany

Patricia Tonin

Canada

Gian Paolo Tonini

Italy

Jeffrey Toretsky

USA

Luigi Tornillo

Switzerland

Piero Tosi

Italy

Paul Townsend

United Kingdom

Gillian Tozer

United Kingdom

Jaume Trapé

Spain

Ruth Travis

United Kingdom

Marialena Trivella

United Kingdom

Kiril Trpkov

Canada

Jau-Yih Tsauo

Taiwan

Ling Ming Tseng

Taiwan

Vivien Tsu

USA

Katsuya Tsuchihara

Japan

Tung Yu Tsui

Germany

Takemasa Tsuji

USA

Allan Tsung

USA

James Turkson

USA

Shelley Tworoger

USA

Muhammad Umer

Pakistan

Naoyuki Umetani

Japan

Marjetka Ursic Vrscaj

Slovenia

Toshikazu Ushijima

Japan

Binnur Üzmez Önal

Turkey

Jaydutt Vadgama

USA

Ratna Vadlamudi

USA

Kannan Vaidyanathan

India

Patricia Valery

Australia

Juan Valle

United Kingdom

Elske Van Den Akker

Netherlands

Twan Van Den Beucken

Netherlands

Ate Van Der Gaast

Netherlands

Petra Van Der Groep

Netherlands

Theodorus Van Der Kwast

Canada

Carolien Van Deurzen

Netherlands

Toon Van Gorp

Netherlands

Willem Herbert Van Harten

Netherlands

Laurens Van Meeteren

Netherlands

Carel Van Noesel

Netherlands

Birgitt Van Oorschot

Germany

Bas Van Rhijn

Netherlands

Maurice Van Steeensel

Netherlands

Dannis Van Vuurden

Netherlands

Jean-Marc Vanacker

France

Barbara Vanderhyden

Canada

Yann Vano

France

Marileila Varella-Garcia

USA

Ana Vargas

Australia

Yogesh Vashist

Germany

Erik Vassella

Switzerland

Gareth Veal

United Arab Emirates

Juergen Veeck

Germany

Irma Verdonck-De Leeuw

Netherlands

Marilena Vered

Israel

Roel Verhaak

USA

Helena Verkooijen

Netherlands

Rajeev Vibhakar

USA

Nadarajah Vigneswaran

USA

Ester Vilaprinyo

Spain

Raffaella Villa

Italy

Antonio Villalobo

Spain

Adam Vilmar

Denmark

Francesc Vinals

Spain

Fabrizio Vinante

Italy

Elizabeth Vincan

Australia

Yana Vinogradova

United Kingdom

Loes Visser

Netherlands

Satish Viswanath

USA

Umberto Vitolo

Italy

Rui Vitorino

Portugal

Adele Vivacqua

Italy

Francisco Vizoso

Spain

Antonia Vlahou

Greece

Erina Vlashi

USA

Tatyana Vlaykova

Bulgaria

Martina Vockerodt

United Kingdom

Pavel Vodicka

Czech Republic

Iris Vogelaar

Netherlands

Anja Von Heydebreck

Germany

Mia Voordeckers

Belgium

Dirk Vordermark

Germany

Markku Voutilainen

Finland

Adina Vultur

USA

Nadarajen Vydelingum

USA

Masaru Wakatsuki

Japan

Todd Waldman

USA

Jens Waldmann

Germany

Christine Walsh

USA

Naomi Walsh

Ireland

Chunmei Wang

USA

Han-I Wang

United Kingdom

Jaw-Yuan Wang

Taiwan

Jianbing Wang

USA

Jing Wang

China

Longgui Wang

USA

Peizhong Wang

Canada

Qianben Wang

USA

Rong Fu Wang

China

Rui Wang

China

Shuyi Wang

China

Xin Shelley Wang

USA

Yaning Wang

USA

Janindra Warusavitarne

United Kingdom

Kounosuke Watabe

USA

Masatoshi Watanabe

Japan

Joanna Watson

United Kingdom

Rebecca Watters

USA

Achim Weber

Switzerland

Christopher Weber

USA

Kristy Weber

USA

Ashani Weeraratna

USA

Henning Wege

Germany

Lixin Wei

China

Qingyi Wei

USA

William Wei

China

Chen Weichang

China

Andrew Weickhardt

USA

Hannah Weir

USA

Frank Ulrich Weiss

Germany

Alessandro Weisz

Italy

Wu Weizhong

China

Scott Welford

USA

Grit Welzel

Germany

Patrick Wen

USA

Zhe-Sheng Wen

China

Ye Wencai

China

Nicolas Wentzensen

USA

Haim Werner

Israel

Cynthia Wetmore

USA

Hayley Whitaker

United Kingdom

James Whiteford

United Kingdom

Vicki Whitehall

Australia

Theresa Whiteside

USA

Piotr Widlak

Poland

Gro Wiedswang

Norway

Joseph Wiemels

USA

Charlotte Wilhelm-Benartzi

United Kingdom

Ann Williams

United Kingdom

Kaye Williams

United Kingdom

Carmen Wilson

USA

Robin Wilson

USA

William Wilson

New Zealand

Joerg Wilting

Germany

Aung Ko Win

Australia

Reinhard Windhager

Austria

Steve Wing

USA

Sebastiaan Winkler

United Kingdom

Zoe Winters

United Kingdom

Olaf Witt

Germany

Thomas Witzig

USA

Jennifer Wo

USA

Alice Wong

Hong Kong

Nick Wong

Australia

Laura Woods

United Kingdom

Frances Wright

Canada

Jiangping Wu

Canada

Lili Wu

China

Bing Xia

China

Jinglin Xia

China

Tingyi Xia

China

Chen Xiaolan

China

Xing Xie

China

Kecheng Xu

China

Liang Xu

USA

Zhi-Xiang Xu

USA

Yasuhide Yamada

Japan

Hideki Yamaguchi

Japan

Hiroshi Yamaguchi

Japan

Naoko Yamamoto

Japan

Hiroko Yamashita

Japan

Keishi Yamashita

Japan

Hideya Yamazaki

Japan

Chunhong Yan

USA

Xiaojun Yan

China

Nozomu Yanaihara

Japan

Jianhua Yang

USA

Li-Ye Yang

China

Sherry Yang

USA

Shung-Haur Yang

Taiwan

Xianghong Yang

China

Yongping Yang

China

Dengfu Yao

China

Song Yao

USA

Timothy Yap

United Kingdom

Pedram Yazdan

USA

Eugenia Yazlovitskaya

USA

Xiaofei Ye

China

Chia Jui Yen

Taiwan

Gow-Chin Yen

Taiwan

Joo Mi Yi

Korea, South

Qing Yi

USA

Zhihua Yin

China

Songmin Ying

United Kingdom

Tsung-Ho Ying

Taiwan

Yoshihito Yokoyama

Japan

Toshitaka Yoshii

Japan

Greg Yothers

USA

Wei-Cheng You

China

Danny Youlden

Australia

Leonie Young

Ireland

Aiping Yu

China

Jian Yu

USA

Li-Rong Yu

USA

Mansoo Yu

USA

Onchee Yu

USA

Xue Qin Yu

Australia

Yanlin Yu

USA

Yinhua Yu

China

Gelareh Zadeh

Canada

Kurt S. Zaenker

Germany

Ian Zajac

Australia

Mahvash Zakikhani

Canada

Gerard Zalcman

France

Rita Zamarchi

Italy

Maurizio Zanetti

USA

Alfeu Zanotto-Filho

Brazil

Luciano Zardi

Italy

Izabela Zawlik

Poland

Robert Zeillinger

Austria

Changqing Zeng

China

Nikolajs Zeps

Australia

Ming Zhang

USA

Mingfeng Zhang

USA

Qingying Zhang

China

Rihua Zhang

China

Yifan Zhang

China

Zhengmao Zhang

USA

Zhi-Ming Zhang

China

Zilai Zhang

USA

Kuai-Le Zhao

China

Shu Zheng

China

Baosen Zhou

China

Yifeng Zhou

China

Tao Zhu

China

Daniel Zicha

United Kingdom

Ariana Znaor

Croatia

Wainer Zoli

Italy

Massimo Zollo

Italy

Stanley Zucker

USA

Jessica Zucman-Rossi

France

Valentina Zuco

Italy

Martin Zweifel

United Kingdom

## Pre-publication history

The pre-publication history for this paper can be accessed here:

http://www.biomedcentral.com/1471-2407/13/186/prepub

